# Supplementary material for: Molecular Detection of Venous Thrombosis in Mouse Models Using SPECT/CT
Source: Biomolecules. 2022 Jun 13;12(6):829. doi: 10.3390/biom12060829 (PMC9221411; doi:10.3390/biom12060829)
Supplement: Supplementary file 1 [file biomolecules-12-00829-s001.zip › biomolecules-1753761-supplementary.pdf]

Supporting information

Molecular detection of venous thrombosis in mouse models using SPECT/CT

Annemiek Dickhout, Pieter van de Vijver, Nicole Bitsch, Stefan J van Hoof, Stella L. G. D. Thomassen, Steffen Massberg, Peter Timmerman, Frank Verhaegen, Rory R. Koenen, Ingrid Dijkgraaf, Tilman M. Hackeng

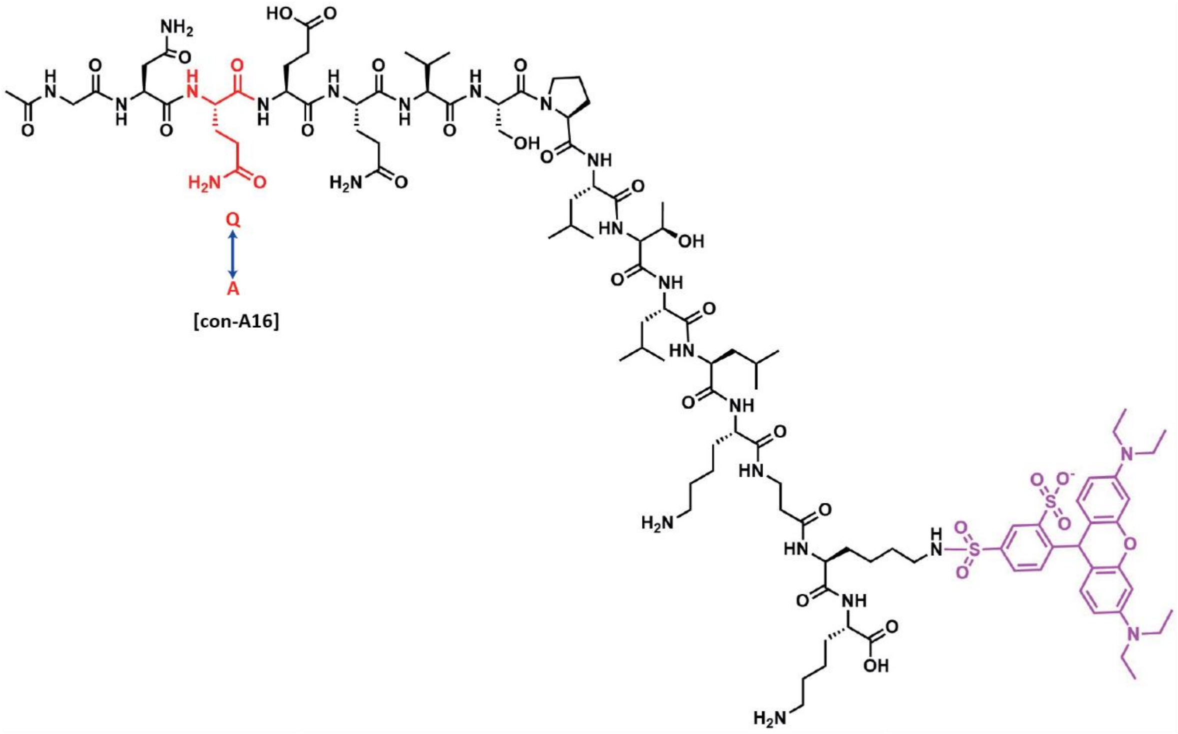

Figure S1. Schematic representation of A16-LisB. Substitution of glutamine to alanine (Q3 --> A3) leads to a control probe (con-A16) which is not bound to fibrin.

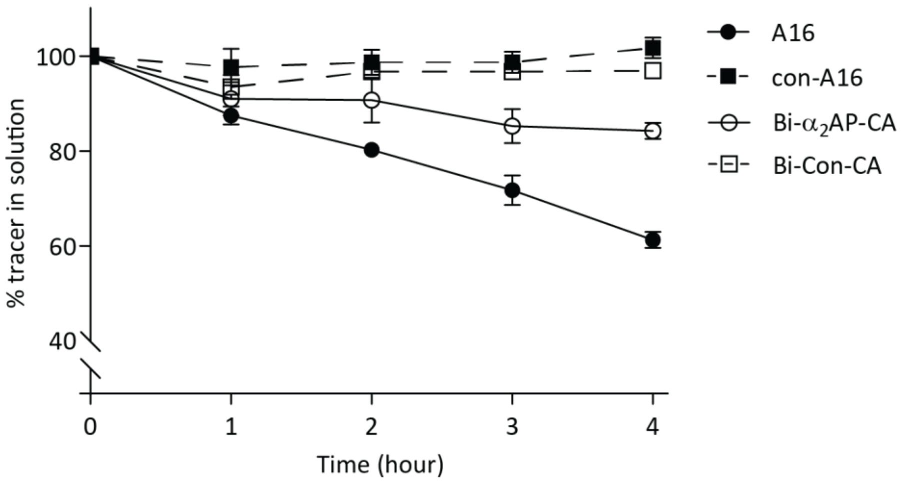

Figure S2. *In vitro* probe depletion. Human plasma was allowed to form thrombi *in vitro* at 37 °C for 90 minutes. Rhodamine-labelled probes (15 µM) were added to human thrombi. OD (570 nm) in solution was measured after 1, 2, 3 and 4 hours, as a measure of probe incorporation in thrombi. Dots represent mean ±SD, n=4.

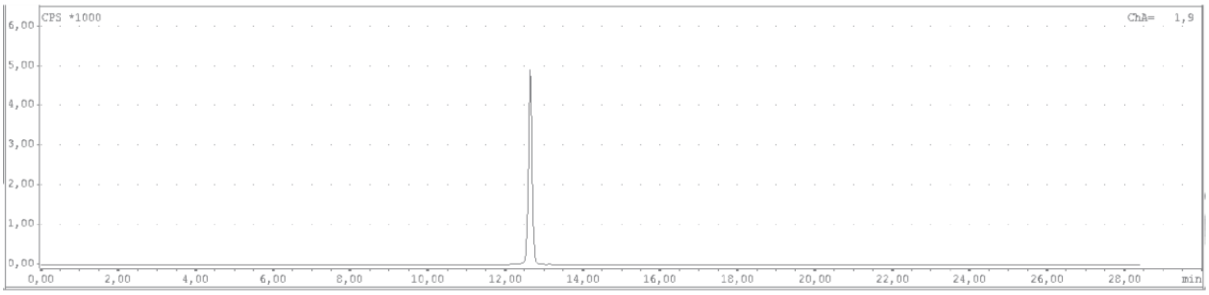

Figure S3. Representative radio-chromatogram of ^{111}In-A16 after radio-HPLC analysis.

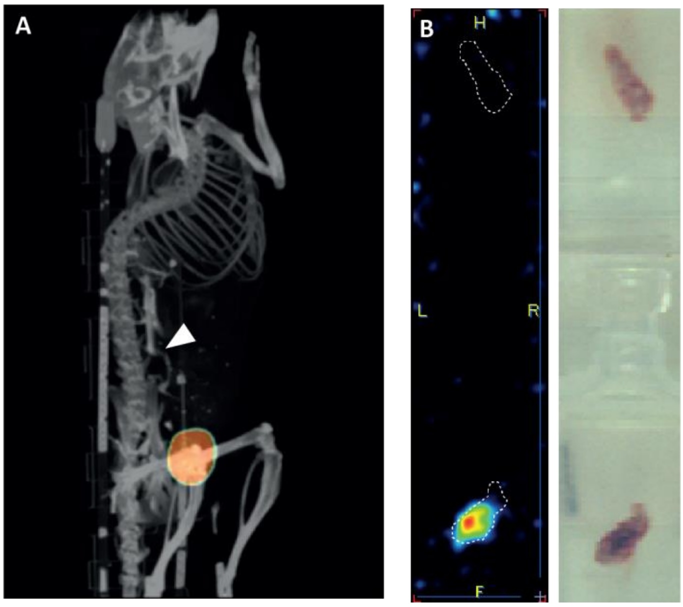

Figure S4. (A) Representative SPECT/CT overlay of a mouse 6 hr after IVC ligation, injected with ^{111}In-DTPA-A16. High uptake is seen in the bladder, while lack of contrast (indicated with arrowhead) indicating thrombus formation. No SPECT signal is visible. (B) *Ex vivo* SPECT scan of a sham-surgery IVC (top dotted line) and a thrombus excised from the mouse in A (bottom dotted line) injected with ^{111}In-A16.
